# New Generation of Resistant Sugar Beet Varieties for Advanced Integrated Management of Cercospora Leaf Spot in Central Europe

**DOI:** 10.3389/fpls.2018.00222

**Published:** 2018-02-27

**Authors:** Johannes Vogel, Christine Kenter, Carsten Holst, Bernward Märländer

**Affiliations:** ^1^Institute of Sugar Beet Research at the University of Göttingen, Göttingen, Germany; ^2^Department of Agricultural Economics and Rural Development, University of Göttingen, Göttingen, Germany

**Keywords:** *Cercospora beticola*, sugar beet, variety trials, resistance, breeding progress, sugar beet yield, yield penalty, economic performance

## Abstract

Cercospora leaf spot (CLS) epidemics in sugar beet have been increasing in recent years causing higher use of fungicides. Concomitantly, the availability of effective fungicides is at risk because of resistance development in the fungus, the lack of new active ingredients as well as restrictive approval practices. A key option for an integrated management of CLS is cultivation of resistant varieties. Because of the yield penalty in resistant varieties, acceptance in commercial practice so far has been low. The aim of our study was to characterize recent sugar beet varieties registered in Germany in terms of resistance and tolerance to CLS and their value for integrated pest management. The genetic basis of CLS resistance in varieties is protected by intellectual property rights even after variety registration and not open to the public due to economic competition. To gain reliable data for cultivation, varieties have to be tested for their resistance traits under field conditions at varying levels of infection with *Cercospora beticola*. In collaboration with variety related stakeholders, 15 sugar beet varieties were tested in 49 field trials in Germany from 2014 to 2016 for their yield response to CLS. The trials were set up in a split-plot design with and without infection (i.e., with and without fungicide). The classification of varietal reaction to CLS is based on symptomatic leaf area (susceptibility) and the resulting relative yield loss (tolerance). Since the relation between both parameters varied among varieties, it was used as an additional parameter to describe tolerance. On this basis, three groups of varieties were identified. They can be characterized as a susceptible, a resistant and a presumably tolerant cluster. A comparison of the data with an older dataset originating from 2009 to 2011 revealed that yield performance of recent varieties with resistance to *C. beticola* caught up with susceptible varieties due to breeding progress. They showed no yield penalty in the absence of the disease and better economic performance than susceptible varieties. It is assumed that these varieties will allow a substantial reduction of fungicide use for an advanced integrated pest management under central European conditions.

## Introduction

Cercospora leaf spot (CLS) caused by the fungus *Cercospora beticola* Sacc. is the most widespread and most damaging foliar disease in sugar beet (*Beta vulgaris* L.) worldwide (Skaracis et al., 2010). Yield losses up to 50% and inferior processing quality caused by CLS have been reported (Wolf et al., 1998; Rossi et al., 2000). In recent years, 60–90% of the German sugar beet area was infested by *C. beticola*, whereas other pathogens (*Erysiphe betae, Uromyces betae*, and *Ramularia beticola*) occurring on less than 20% had a significantly lower economic importance (Brendler et al., 2008; Vasel et al., 2013).

The area infested with CLS has steadily enlarged from the southern and western part to the north and east of Germany (Buhre et al., 2014). Model calculations for different regions forecast even more favorable conditions for the fungus in the future resulting in an earlier occurrence of CLS, and increasing use of fungicides is discussed (Richerzhagen et al., 2011; Kremer et al., 2016). This development contrasts with the public request to reduce pesticide use and with the principles of integrated pest management. They are implemented by European law, stating pesticide use to be reduced to the necessary minimum (EU, 2009). To meet this demand, infection threshold values for fungicide application were developed (Wolf and Verreet, 2002; Lang, 2005) and field monitoring as well as forecasting models are employed to derive site specific control strategies (Racca et al., 2004). In the past, one fungicide application was sufficient in most cases to control CLS under German conditions, but three necessary applications have been reported as well (Buhre et al., 2014; Roßberg et al., 2017).

The widespread use of fungicides and the consequent selection pressure on *C. beticola* caused the development of resistances against fungicides with different modes of action (Varrelmann and Märländer, 2017). Already in the 1970s and 1980s, resistance against benzimidazoles was observed in southern Europe and the United States. Benzimidazole fungicides were mainly replaced by triazoles and strobilurins, which in turn led to a shift in the sensitivity of *C. populations* to triazoles and to resistance against strobilurines as summarized by Karaoglanidis and Ioannidis (2010).

The development of fungicide resistances underlines the necessity of an integrated management of CLS relying on other means beyond fungicides. A key factor is breeding for resistance against *C. beticola* in sugar beet. Varietal resistance against pathogens often comes along with a yield penalty in the absence of the disease (Brown, 2002). This was also found in several studies with sugar beet (e.g., Miller et al., 1994; Mittler et al., 2004; Kaiser et al., 2010; Gummert et al., 2015). Breeding of resistant varieties with high yield performance even without or under low infection pressure is crucial for acceptance in commercial practice. Whereas resistance describes the quality to hinder the development of a pathogen, the ability to produce high yield even under severe infection is called tolerance (Agrios, 2005). The resistance to CLS in sugar beet is quantitatively inherited and based on at least 4 to 5 major resistance genes and thus expressed gradually (Smith and Gaskill, 1970; Weiland and Koch, 2004). The genetic basis of CLS resistance in varieties is protected by intellectual property rights even after registration, i.e., not open to the public due to economic competition. To gain reliable data for cultivation, varieties have to be tested for their resistance traits under field conditions. In Germany, sugar beet varieties are tested in nationwide trials with two fungicide levels. The plots are either non-treated or fungicides are applied repeatedly to keep the crop as healthy as possible for a *ceteris paribus* comparison of varietal performance with and without foliar diseases (Ossenkop et al., 2005).

To describe the varieties according to their reaction toward CLS, two parameters are used. The first one is the infection of the leaves with CLS based on a grading of disease severity (DS) before harvest in the level without fungicide (BSA, 2000). It indicates the level of resistance/susceptibility toward CLS. The second parameter is yield loss caused by CLS. It is calculated as the relative difference in white sugar yield (WSY) between the non-treated and healthy fungicide levels and is supposed to describe the tolerance toward CLS (Ossenkop et al., 2005). As resistance against CLS maintains photosynthetic leaf area and thereby reduces yield loss (Rossi et al., 2000), it has been a matter of discussion, whether reduced yield loss can be attributed to tolerance traits or not (Ossenkop et al., 2005; Kaiser et al., 2010). Consequently, it has to be evaluated in more detail how CLS resistance and tolerance are connected in sugar beet varieties.

The aim of the present study was (i) to identify parameters to characterize resistance and tolerance toward CLS in sugar beet varieties, (ii) to distinguish variety groups according to their reaction toward CLS, (iii) to assess whether the yield penalty in resistant varieties has changed in recent years, and (iv) to describe consequences for beet cultivation and integrated pest management.

## Materials and Methods

### Field Trials

The data originated from national variety trials with sugarbeet in Germany. Two 3-year datasets from 2014–2016 (trial series 1) and 2009–2011 (trial series 2) at 45 and 49 environments (i.e., location × year), respectively, were analyzed. The 49 trials in series 2 were part of a bigger dataset analyzed earlier by Gummert et al. (2015). All trials were run according to the official guidelines for the implementation of agricultural variety trials (BSA, 2000). Sugar beets were sown between beginning of March and end of April in three-row plots of 10.8–12.0 m^2^. As plant density may cause unintended variance in root yield, the plots were manually thinned after field emergence to a density of 80,000–90,000 plants ha^-1^.

The trial setup was a randomized split-plot design with two replications. The main-plot factor was fungicide strategy and the subplot factor was variety. The two fungicide strategies included a treatment without fungicide application (‘non-treated’) and a treatment with fungicide application aiming to keep the sugar beets as healthy as possible (‘healthy’). This setup allows the comparison of variety performance with and without leaf diseases. Fungicide application started at the onset of first symptoms of foliar diseases in the susceptible varieties and was repeated if symptoms recurred. The last application was timed to comply with the pre-harvest interval (21–35 days depending on the product) at the earliest possible harvest date. The fungicides applied were chosen site-specifically. They mainly belonged to the groups of triazoles and strobilurines. Even though fungicides were sprayed regularly, foliar diseases could occasionally occur. As the disease level remained rather low, the plots were considered as healthy (Gummert et al., 2015).

The trials were harvested between mid-September and beginning of November. Root yield and quality were determined at the local sugar factories. The beets were weighed after washing and processed to beet brei. The brei samples were analyzed for sucrose, potassium, sodium, and amino-nitrogen with automatic beet laboratory systems (Venema Installations, Eeemshaven, Netherlands or Anton Paar OptoTec GmbH, Seelze, Germany) according to standardized procedures (Hoffmann, 2006). WSY as the key indicator of variety performance was calculated from root yield and quality parameters according to German standard equations (Märländer et al., 2003).

### Varieties

In trial series 1 and 2, 15 and 13 varieties were tested, respectively, which represented the varieties available for cultivation in Germany (**Table 1**). Each variety was tested at all environments within one series. Variety ratings for susceptibility to CLS according to the German variety list (BSA, 2011/2016) ranged from 3 to 5 in series 1 and from 2 to 5 in series 2. Tolerance to foliar diseases was calculated as the difference of relative WSY between the healthy and non-treated levels, i.e., the yield loss due to foliar diseases (IfZ, 2011, 2016). The larger the negative value, the less tolerant the variety was.

**Table 1:** Sugar beet varieties tested in national variety trials in Germany 2014–2016 and 2009–2011.

Test period	Variety	ID	Release	Susceptibility	Tolerance
2014–2016	1	1665	2006	4	-4.3
	2	1991	2010	4	-4.9
	3	2056	2011	4	-5.9
	4	2059	2011	5	-7.5
	5	2097	2011	3	-5.9
	6	2148	2012	4	-5.9
	7	2155	2012	4	-7.3
	8	2158	2012	4	-6.7
	9	2192	2012	3	-5.3
	10	2197	2012	4	-5.2
	11	2257	2013	5	-7.6
	12	2301	2013	4	-6.6
	13	2306	2013	4	-5.2
	14	2309	2013	3	-4.8
	15	2313	2013	5	-7.8
2009–2011	1	1665	2006	4	-4.0
	101	1409	2003	4	-3.2
	102	1492	2004	3	-3.6
	103	1560	2005	4	-5.1
	104	1632	2006	4	-5.0
	105	1648	2006	3	-3.8
	106	1718	2007	4	-5.9
	107	1748	2007	5	-3.9
	108	1779	2008	4	-4.3
	109	1802	2008	2	-5.2
	110	1806	2008	4	-4.9
	111	1824	2008	3	-2.5
	112	1830	2008	4	-5.3

*Identification number (ID), year of release and rating of susceptibility to Cercospora leaf spot (CLS) (susceptibility) according to German Federal Plant Variety Office (BSA, 2011/2016): 1 = absent/very low to 9 = very high. Tolerance to foliar diseases (tolerance): relative loss of white sugar yield (WSY) between treatments with and without fungicide; 100 = mean of standard varieties in the level with fungicides (BSA, 2000; IfZ, 2011, 2016).*

### Disease Severity and Classification of Environments

The occurrence of *C. beticola* and other foliar pathogens (*R. beticola, E. betae, U. betae*) was regularly assessed in all trials. DS of each foliar disease was rated by plot on a 1–9 scale (1: no infection, 9: very high infection) at least twice between canopy closure and harvest according to BSA (2000). CLS was the predominant foliar disease in both trial series (data not shown). The CLS rating with the greatest differentiation among varieties (DS_end_) was used for further data analyses (BSA, 2000; Gummert et al., 2015). This was with few exceptions the rating before harvest.

Environments were assigned to levels of infection according to mean DS_end_ of CLS in all varieties in the level without fungicide. Gummert et al. (2015) concluded that two groups of infection levels are sufficient to evaluate variety performance due to marginal differences between environments without or low to medium infection. Environments with DS_end_ < 5 were thus summarized in one group with no/low infection and DS_end_ ≥ 5 was regarded as high infection (**Table 2**). This was in line with studies by Uphoff (2011) and Hoberg et al. (2015). Environments without CLS but with other foliar diseases were excluded from the dataset. Environments with CLS and further foliar diseases were also excluded if a fungicide effect was found which was related to high ratings of other foliar diseases than CLS.

**Table 2:** Classification of environments according to mean disease severity (DS) of Cercospora leaf spot (15 varieties in 2014–2016, 13 varieties in 2009–2010) without fungicide application.

	Disease severity of Cercospora leaf spot	
Year	Low (<5)	High (≥5)
	No. of environments	
2014	10	5
2015	11	4
2016	9	6
2014–2016	30	15
2009	5	6
2010	20	2
2011	11	5
2009–2011	36	13

*National variety trials in Germany, DS rating according to BSA (2000).*

### Statistical Evaluation

Statistical analysis was carried out with SAS Desktop-Version 9.4 (SAS Institute, Inc., Cary, NC, United States). The MIXED procedure was applied for ANOVA of WSY with *post hoc* Tukey-Test and estimation of variance components. To describe the relation of DS_end_ and relative loss of WSY, Spearman’s rank correlation coefficient was calculated with the CORR procedure and regression analysis and calculation of residuals was made with the REG procedure.

DS_end_ and relative loss of WSY were used as cluster-building variables in a cluster analysis. The aim was grouping of the varieties, i.e., to reveal groups with high similarities within and as many differences as possible between clusters. The SAS procedure DISTANCE was used to calculate Euclidian distances for the distance matrix. For cluster generation, the average linkage method was used with the procedure CLUSTER considering the mean distances between the members of two different clusters. The resulting differences between clusters were visualized in a dendrogram. Distances from 0.0 to 0.1 were considered to show very high analogy, from 0.1 to 0.3 high, from 0.3 to 0.5 average, and from 0.5 to 0.7 low analogy between the groups. No analogy was assumed for distances ≥0.7 (Hoberg et al., 2015).

### Economical Evaluation

Economic performance of the different variety clusters was assessed with management accounting using (a) yield and quality data from the 2014–2016 field trials, (b) beet prizes 2017 in 1-year contract of Nordzucker (2016), (c) input data for seeds, fertilizers and plant protection products from a farm survey in Germany in 2012–2014 (Stockfisch et al., 2013), (d) mean costs of seeds at the sugar companies Südzucker AG (BISZ, 2017), Nordzucker AG (Ewers, personal communication) and Pfeifer & Langen GmbH & Co. KG (LIZ, 2017), mean costs for fertilizers in Lower Saxony January–March 2017 (Landwirtschaftskammer Niedersachsen, 2017) and mean costs for plant protection products at agricultural dealers (AGRAVIS Raiffeisen AG, Münster and Hanover, and BayWa AG, Munich), (e) farm business management data bases (KTBL, 2017; Uppenkamp and Nacke, 2017) to estimate labor and machinery costs of fungicide application based on the aforementioned German farm survey. The number of fungicide applications according to the threshold system (Wolf and Verreet, 2002; Lang, 2005) was assumed according to variety cluster and disease pressure (**Table 3**).

**Table 3:** Number of fungicide applications according to the threshold system (Wolf and Verreet, 2002; Lang, 2005) in sugar beet varieties susceptible, tolerant, and resistant to *Cercospora beticola* at environments with disease severity (DS) <5 (low) and DS ≥5 (high); rating according to BSA (2000).

Variety type	Disease severity of CLS	
	Low	High
Susceptible (A)	1	3
Tolerant (B)	1	3
Resistant (C)	1	2

## Results

### White Sugar Yield

White sugar yield in the 2014–2016 trials was significantly influenced by environment, fungicide level, variety and their interactions (**Table 4**). Fungicide level and environment had the strongest influence whereas the effect of variety was much smaller and on a similar level with the environment × fungicide interaction. All further interactions were of minor relevance.

**Table 4:** Analysis of variance for factors influencing white sugar yield of 15 sugar beet varieties tested at 45 environments in Germany 2014–2016.

Effect	DF	Sum of squares	Mean square	*F*-Value
Environment	44	13049.4	296.6	584.0 ^∗∗∗^
Fungicide	1	585.3	585.3	1152.5 ^∗∗∗^
Variety	14	276.6	19.8	38.9 ^∗∗∗^
Environment × fungicide	44	481.4	10.9	21.6 ^∗∗∗^
Environment × variety	616	752.9	1.2	2.4 ^∗∗∗^
Fungicide × variety	14	25.2	1,8	3.5 ^∗∗∗^
Environment × fungicide × variety	616	321.8	0.5	1.0 n.s.
Replication (environment)	45	204.6	4.5	9.0 ^∗∗∗^
Error	1305	662.7	0.5	
Corrected sum	2699	16359.9		

*CV, coefficient of variance; DF, degrees of freedom; ^∗∗∗^*P* ≤ 0.001; n.s., not significant.*

The estimation of variance components for the different levels of CLS infection and fungicide confirmed the dominant influence of environment on WSY (**Table 5**). With increasing disease pressure, the effect of variety significantly increased from 1.6% under healthy conditions at low CLS infection to 4.0% in the non-treated level at high infection. Similarly, the environment × variety interaction increased from 0.0 to 3.8%.

**Table 5:** Estimation of variance components (%) for factors influencing white sugar yield of sugar beet at low and high disease severity of Cercospora leaf spot and two fungicide levels (non-treated/healthy); 15 varieties tested at 45 environments in Germany 2014–2016.

	Low infection (*n* = 30)		High infection (*n* = 15)	
Fungicide level	Healthy	Non-treated	Healthy	Non-treated
Environment	87.6 a	87.3 a	81.7 a	80.4 a
Variety	1.6 b	1.8 ab	2.0 ab	4.0 a
Environment × variety	0.0 c	0.9 b	4.5 a	3.8 a
Error	11.0 a	9.1 b	11.8 a	11.8 a

*Different letters indicate significant differences within each row (Tukey-Test, *P* ≤ 0.05).*

**FIGURE 1:**
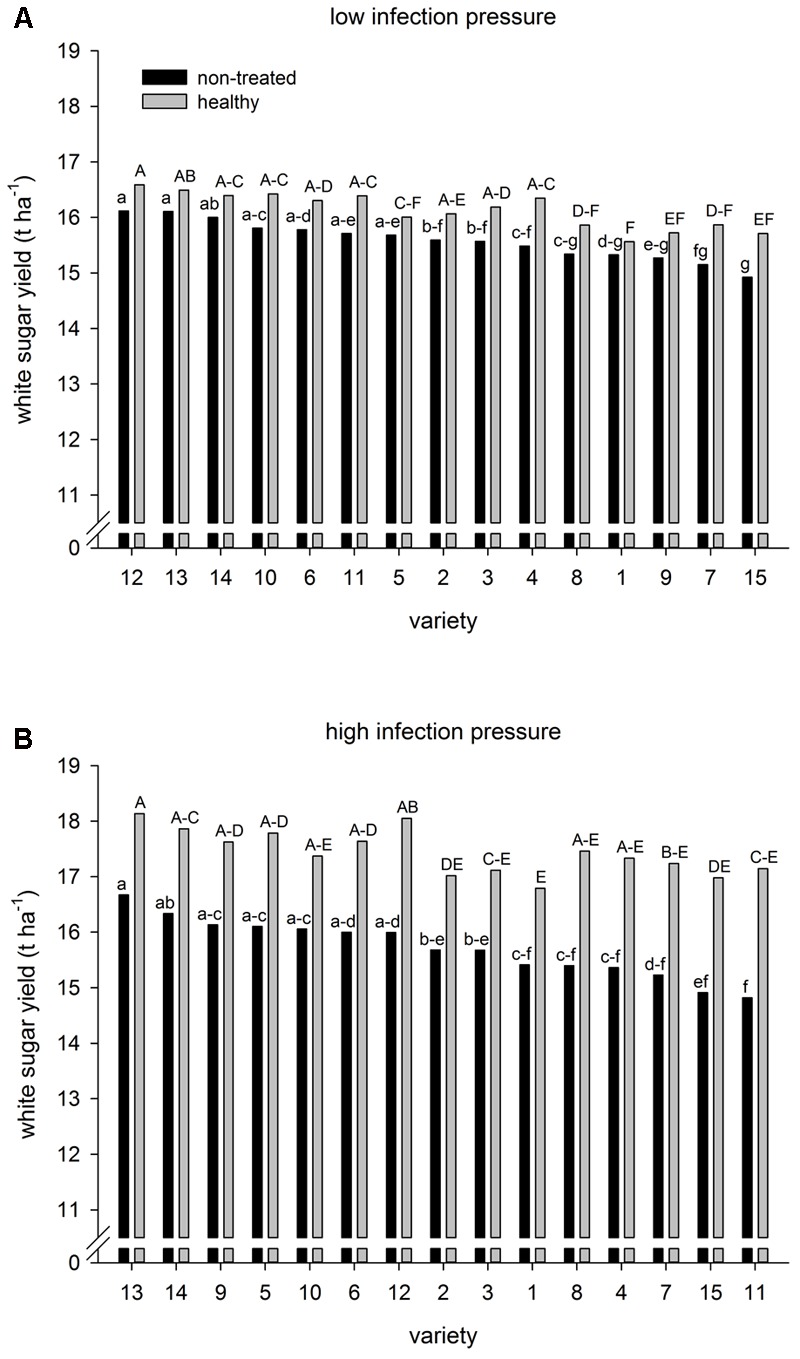
White sugar yield of sugar beet varieties at environments with **(A)** low and **(B)** high infection with *Cercospora beticola* at two fungicide levels (non-treated and healthy). 30 and 15 environments in Germany, 2014–2016. Different lower case letters indicate significant differences in the non-treated level; different upper case letters indicate significant difference in the healthy level (Tukey-Test, *P* ≤ 0.05).

Under low infection, mean WSY across varieties was 15.59 t ha^-1^ in the non-treated and 16.12 t ha^-1^ in the healthy level (**Figure 1A**). The difference between the two fungicide levels ranged from 0.24 to 0.86 t ha^-1^ among varieties. Changes in the variety ranking between non-treated and healthy were relatively small. Under high infection, mean WSY was 15.72 t ha^-1^ in the non-treated and 17.44 t ha^-1^ in the healthy level (**Figure 1B**). The varietal difference between both levels was 1.04–2.52 t ha^-1^, i.e., the range was wider than under low infection causing greater changes in the variety ranking between fungicide levels. These changes were greatest in varieties 1 and 2, which ranked lower in the healthy than in the non-treated level, and varieties 8, 11, and 12 reacting vice versa. Comparing the levels of CLS infection, even greater changes in variety ranking occurred. Varieties 9 and 11, e.g., were placed 13^th^ and 6^th^ under low infection and 3^rd^ and 15^th^ under high infection (non-treated). By contrast, other varieties showed high yield stability, namely varieties 13 and 14.

### Disease Loss Relation

**FIGURE 2:**
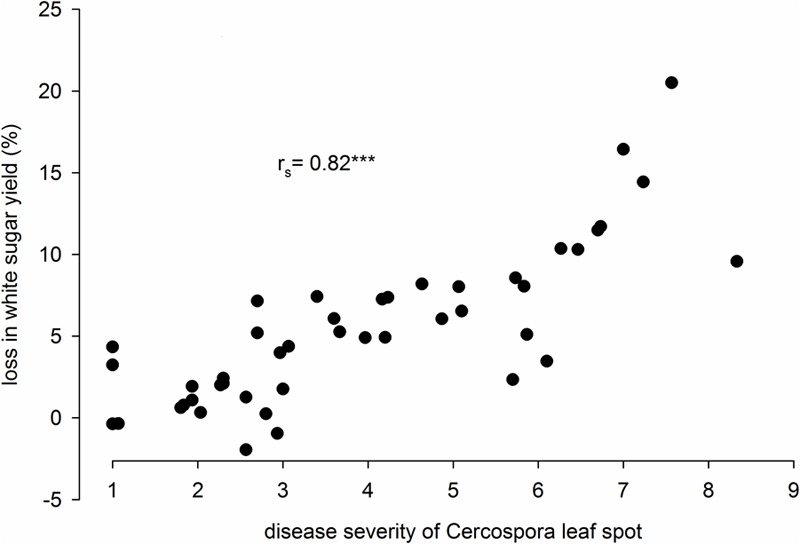
Disease severity (DS) of Cercospora leaf spot and relative loss in white sugar yield (WSY) in 45 environments in Germany, 2014–2016; mean of 15 varieties. Relative loss in white sugar yield (WSY) is the yield difference between healthy and non-treated fungicide levels as percentage of WSY in the healthy level. DS was rated in the non-treated plots on a 1–9 scale (1: no infection, 9: very high infection; BSA, 2000); r_s_ = Spearman’s rank correlation coefficient, ^∗∗∗^*P* ≤ 0.001.

In the 2014–2016 trials, mean DS_end_ in the non-treated fungicide level varied from 1.0 to 8.3 among environments covering almost the whole 1–9 scale (**Figure 2**). The corresponding loss in WSY ranged between -2 and 21% and significantly increased with increasing DS_end_.

**FIGURE 3:**
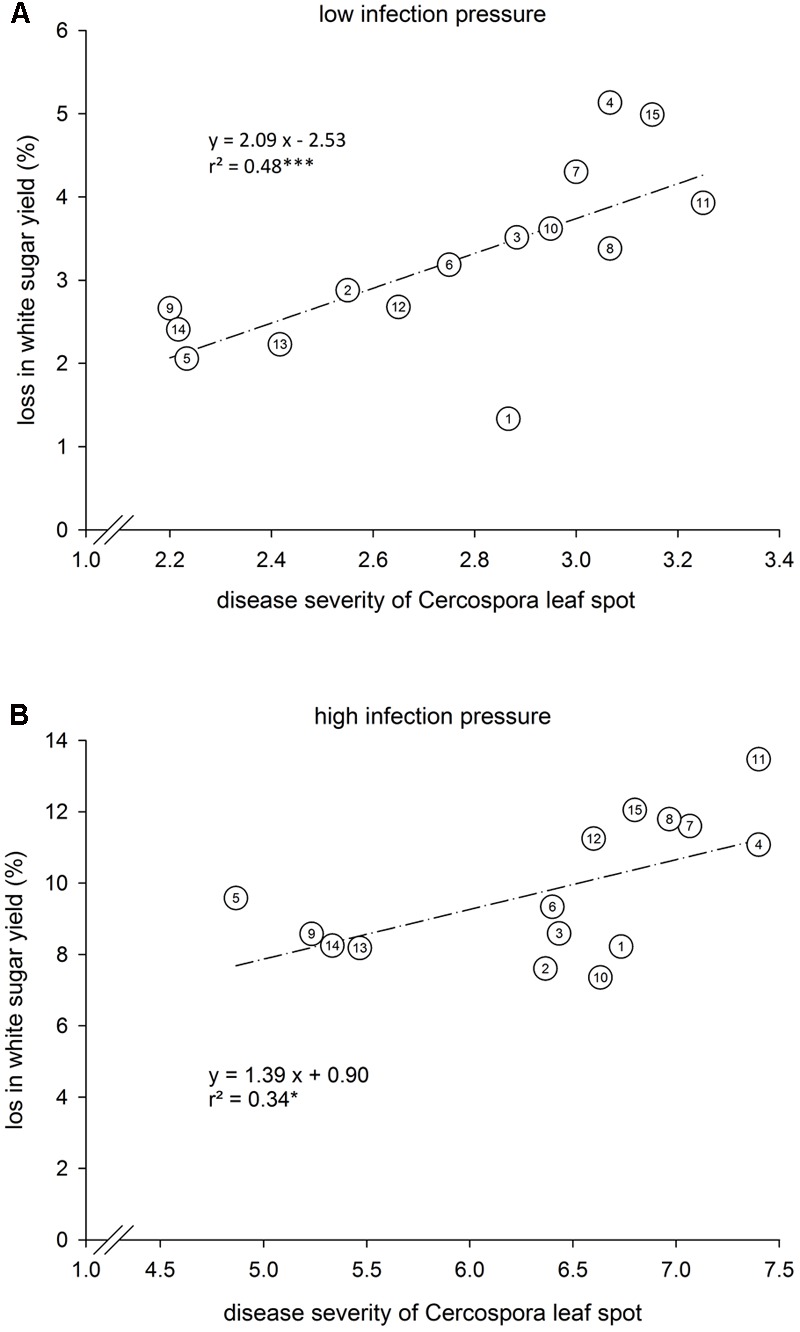
Disease severity of Cercospora leaf spot (CLS) and relative loss of white sugar yield (WSY) in 15 sugar beet varieties tested at **(A)** 30 environments with low infection with CLS and **(B)** 15 environments with high infection with CLS; Germany, 2014–2016 under high infection in the non-treated level. Relative loss in WSY is the yield difference between healthy and non-treated fungicide levels as percentage of WSY in the healthy level. DS was rated in the non-treated plots on a 1–9 scale (1: no infection, 9: very high infection; BSA, 2000). ^∗^*P* ≤ 0.05 and ^∗∗∗^*P* ≤ 0.001.

**FIGURE 4:**
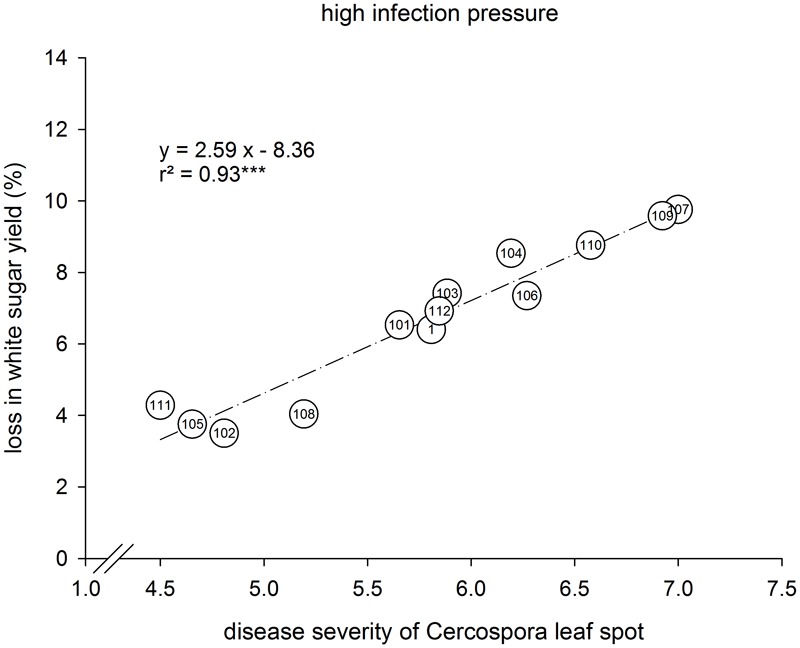
Disease severity of Cercospora leaf spot (CLS) and relative loss of white sugar yield (WSY) in 13 sugar beet varieties tested at 13 environments with high infection with CLS; Germany, 2009–2011. Relative loss in WSY is the yield difference between healthy and non-treated fungicide levels as percentage of WSY in the healthy level. DS was rated in the non-treated plots on a 1–9 scale (1: no infection, 9: very high infection; BSA, 2000). ^∗∗∗^*P* ≤ 0.05 and 0.001.

The disease loss relation for the different varieties was separately assessed for low and high CLS infection (**Figure 3**). Under low infection with DS_end_ ranging from 2.2–3.3, yield loss was 1.3–5.1% (**Figure 3A**). Many varieties differed significantly inDS_end_. Significant differences in relative yield loss only occurred between variety 1 and varieties 4 and 15. Under high infection, DS_end_ was 4.9–7.4 (**Figure 3B**). The corresponding yield loss ranged from 7.4 to 13.5% WSY being significantly lower in varieties 2 and 10 than in variety 11. In the 2009–2011 trials, a closer disease loss relation under high infection was found than in 2014–2016 (**Figure 4**). DS_end_ ranged from 4.5 to 7.0 among varieties. Yield loss was 3.5–9.8% and thus lower than in 2014–2016.

### Variety Grouping

**FIGURE 5:**
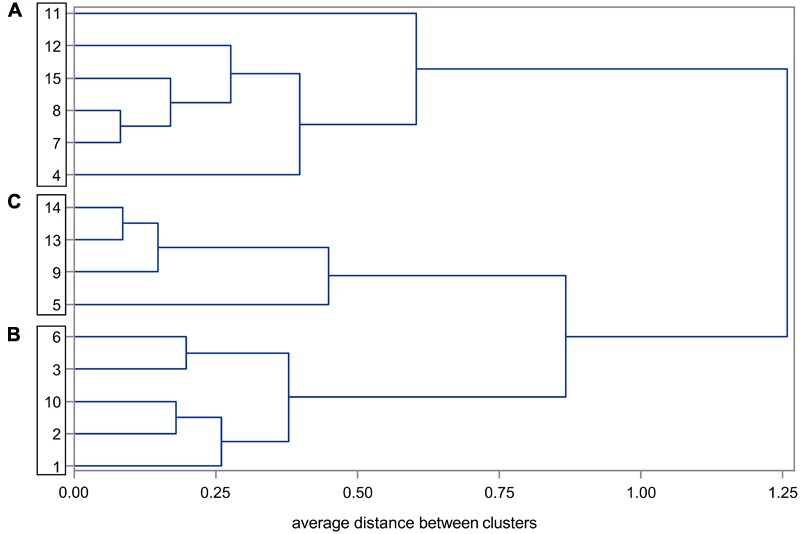
Dendrogram of sugar beet varieties obtained through average linkage cluster analysis based on disease severity of Cercospora leaf spot and relative loss of white sugar yield (WSY). 15 varieties tested at 15 environments in Germany, 2014–2016 under high infection in the non-treated level. Relative loss in WSY is the yield difference between healthy and non-treated fungicide levels as percentage of WSY in the healthy level. **(A–C)** Denote clusters with an average distance of 0.7.

Based on DS_end_ of CLS and relative loss of WSY as cluster-building variables, in trial series 1 (2014–2016), three groups of varieties (clusters A, B, C) were distinguished at an average distance of 0.7 between clusters (**Figure 5**). Average distances within clusters A, B and C were 0.51, 0.40, and 0.55. For series 2 (2009–2011), three clusters (a, b, c) were identified as well (not shown).

Significant differences between the variety clusters were determined in both trial series (**Table 6**). In the 2014–2016 trials, clusters A and C differed in all traits except for WSY in either fungicide level under low infection (**Table 6A**), cluster B was intermediate. Under high infection, the ranking for WSY was C > B > A in the non-treated and C > B = A in the healthy level with relative loss of WSY being considerably higher (8.2–11.9%) than under low infection (2.3–4.1%). In the 2009–2011 trials, differences between the clusters were less distinct (**Table 6B**). Relative loss of WSY was 2.3–3.1% under low infection and 3.9–8.8% under high infection and thus lower than in series 1.

**Table 6:** Different traits of three clusters (for details see **Figure 5**) of sugar beet varieties tested at **(A)** 45 environments in Germany, 2014–2016 and **(B)** 49 environments in Germany, 2009–2011.

(A)				
**Set 1 (45 environments, 15 varieties)**		**Cluster A**	**Cluster B**	**Cluster C**

	Susceptibility to CLS	4.5 a	4.0 a	3.3 b
	Tolerance to foliar diseases	-7.3 a	-5.2 b	-5.3 b
Low infection level (*n* = 30)	Disease severity of CLS	3.0 a	2.7 a	2.2 b
	Relative loss of WSY (%)	4.1 a	2.9 b	2.3 b
	WSY non-treated	15.5	15.6	15.8
	WSY healthy	16.1	16.1	16.2
High infection level (*n* = 15)	Disease severity of CLS	7.0 a	6.5 b	5.2 c
	Relative loss of WSY (%)	11.9 a	8.2 b	8.6 b
	WSY non-treated (t ha^-1^)	15.3 c	15.8 b	16.3 a
	WSY healthy (t ha^-1^)	17.4 b	17.2 b	17.9 a

**(B)**				

**Set 2 (49 environments, 13 varieties)**		**Cluster a**	**Cluster b**	**Cluster c**

	Susceptibility to CLS	3.8	4.0	3.3
	Tolerance to foliar diseases	-5.0	-4.4	-3.6
Low infection level (*n* = 36)	Disease severity of CLS	3.0 a	2.6 b	2.2 c
	Relative loss of WSY (%)	3.1	2.7	2.3
	WSY non-treated	14.4	14.3	14.0
	WSY healthy	14.9	14.8	14.3
High infection level (*n* = 13)	Disease severity of CLS	6.6 a	5.8 b	4.8 c
	Relative loss of WSY (%)	8.8 a	6.8 b	3.9 c
	WSY non-treated (t ha^-1^)	15.3	15.4	15.8
	WSY healthy (t ha^-1^)	16.8	16.6	16.4

*Susceptibility to Cercospora leaf spot (CLS) and tolerance to foliar diseases according to BSA (2011/2016) and IfZ (2011, 2016). Different letters indicate significant differences within each row (Tukey-Test, *P* ≤ 0.05). WSY, white sugar yield.*

### Economic Performance (Trial Series 1)

**FIGURE 6:**
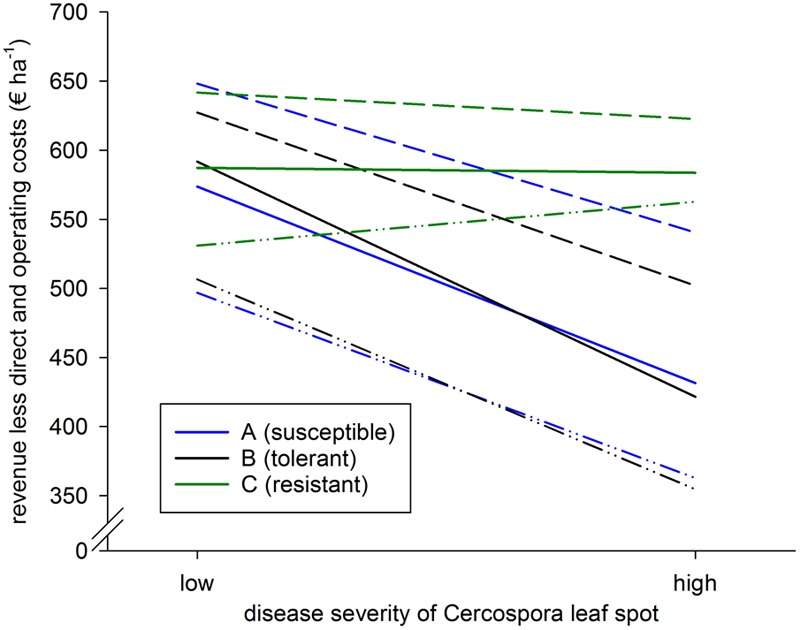
Revenue less direct and operating costs of sugar beet varieties tested in 30 environments with low and 15 environments with high infection with CLS; Germany, 2014–2016. Connecting lines were added to illustrate changes in relative excellence. Susceptible **(A)**, tolerant **(B)**, and resistant **(C)** varieties were clustered according to disease severity of *C. beticola* and yield reaction to the disease. For details see text. Highest and lowest yielding varieties within each group are indicated by dashed and dash-dotted lines, respectively.

Under low infection, mean revenue less direct and operating costs was almost identical for the three clusters A, B, and C (range of 18 Euro ha^-1^; **Figure 6**). Nevertheless, among all varieties, revenue less direct and operating costs of the most profitable and the least profitable variety differed by more than € 150 ha^-1^. However, under high infection, resistant varieties were on average relatively more profitable than tolerant or susceptible varieties. The economic advantage was € 162 ha^-1^ and € 152 ha^-1^, respectively.

## Discussion

The aim of the present study was to identify groups of sugar beet varieties with varying resistance and/or tolerance to CLS within the most recent set of varieties available in Germany. To evaluate breeding progress in resistant varieties and to identify options for an advanced management, the results of 45 national field trials conducted in 2014–2016 (trial series 1) were compared to an older dataset with 49 trials in 2009–2011 (trial series 2).

### Factors Affecting White Sugar Yield

The split-plot design of the field trials with the main factor fungicide made it possible to distinguish between natural infection with CLS and virtually disease free conditions achieved by frequent fungicide application (Ossenkop et al., 2005). Fungicide application had by far the highest influence on WSY of all factors under study, which emphasizes the importance of controlling fungal diseases. The effect of variety was much lower than the effect of environment, as it has been demonstrated before (e.g., Gummert et al., 2015; Hoberg et al., 2015). The environment × fungicide interaction was on a similar level as variety due to the varying severity of CLS infection among environments. Thus, the mean difference between healthy and non-treated conditions was 0.54 t ha^-1^ WSY under low infection, and 1.68 t ha^-1^ under high infection in 2014–2016. Mean WSY was highest in the healthy level under high infection. In Germany, the most severe Cercospora epidemics usually occur in the south where climatic conditions favor the growth of the fungus (Vasel et al., 2013; Gummert et al., 2015). At the same time, WSY is highest in the southern regions where spring temperatures allow early sowing and water supply in summer is high (Kenter et al., 2006; Fuchs et al., 2008). The differences in disease pressure are thus to some extend confounded with regional yield differences, but we do not assume an interaction between regional yield level and fungicide effect.

Estimation of variance components at the two levels of CLS infection under healthy and non-treated conditions confirmed the high environmental effect, which explained >80% of the variance in WSY. Its proportion of variance did not significantly change with increasing pressure of CLS whereas the effect of variety and the environment × variety interaction increased, albeit on a much lower level (<5%). This points to the changes in variety ranking between the levels of infection and fungicide use and is an indication of varying resistance and/or tolerance to CLS. This is in line with results by Gummert et al. (2015).

### Occurrence and Impact of CLS

Heavy CLS infection is necessary to identify resistant varieties, but it does not occur regularly under German climatic conditions (Kaiser and Varrelmann, 2009). Due to the high number of field trials in our study, the whole scale of DS of CLS was covered. High infection occurred in 15 out of 45 trials in the 2014–2016 series, which was sufficient to distinguish different types of varieties (see section “Variety Groups and Yield Performance”).

The loss in WSY caused by CLS increased significantly with increasing level of CLS infection. It increased more rapidly at environments where DS_end_ was above 5, which is presumably due to the non-linear connection of symptomatic leaf area in percent and DS_end_ grading according to the BSA (2000) guidelines. This rating scale was implemented in variety trials for practical reasons to assess resistance in numerous varieties aiming at better discrimination under low infection (BSA, 2000), but it impairs statistical evaluation. In future studies, DS in percent should thus be recorded for a higher accuracy of the regression analysis.

Furthermore, the yield effect of CLS not only depends on the severity, but also on onset time and progress of the epidemics (Wolf and Verreet, 2009). A parameter of the disease progress like the area under the disease progress curve (Shaner and Finney, 1977) thus seems more appropriate to estimate yield losses than single ratings of the disease. Disease progress was not assessed in the present study because the effort for its determination is too high in the high number of official variety trials.

### Variety Groups and Yield Performance

It has been discussed before how resistance and tolerance against CLS in sugar beet varieties can be distinguished (Kaiser et al., 2010). Susceptible varieties express higher DS and loose more photosynthetic leaf area than resistant ones and thus suffer higher relative losses of WSY (Rossi et al., 2000). Ossenkop et al. (2005) thus proposed DS of CLS and relative loss of WSY as describing parameters. In the present study, both DS_end_ and yield loss in dataset 1 varied among the tested varieties indicating varying susceptibility to CLS. This effect was more distinct under high than under low CLS infection confirming the demand for high infection levels for variety characterization (Kaiser and Varrelmann, 2009). The connection of DS_end_ and yield loss was proven at both levels of infection. It was very consistent among the varieties under low infection where merely variety 1 deviated from the regression line. In relation to its DS_end_, it suffered a low relative loss of WSY. Under high infection, several varieties deviated from the regression, i.e., the residuals were larger. In certain varieties (5, 11), yield loss was higher than expected according to DS_end_, in others it was lower (1, 2, 10) pointing to differences in tolerance/sensitivity.

Focusing on the comparative description of single varieties as also done in previous studies (Ossenkop et al., 2005; Kaiser et al., 2010; Gummert et al., 2015) may nevertheless bias the description of resistance or tolerance to CLS by variety traits that are not regarded (e.g., other resistances/tolerances). To avoid this drawback, we carried out a cluster analysis. Cluster A with highest DS_end_ and highest yield loss accordingly had the highest susceptibility to CLS. Cluster C was distinctly less susceptible with lower DS_end_ and lower yield loss, i.e., the varieties within this cluster expressed resistance traits. Cluster B was intermediate. Despite higher DS_end_ than in cluster C, yield loss was lower than expected according to the regression. This effect points to tolerance traits and supports the assumption of Ossenkop et al. (2005) that resistance and tolerance can be distinguished in sugar beet varieties. Cluster B is thus referred to as tolerant.

In the 2009–2011 trials (trial series 2), the cluster analysis resulted in three clusters with different DS_end_ and yield loss as well. By contrast to series 1, there was hardly any deviation from the regression between both parameters under high infection. It is thus concluded that the varieties within this older set represent different degrees of susceptibility/resistance, but there was no intermediate cluster like cluster B in the more recent set. Under low infection, the relative loss in WSY was similar in both trial series (data not shown). Under high infection, it was greater in 2014–2016 than in 2009–2011. Both DS_end_ and yield loss under high infection were similar for cluster B in trial series 1 (referred to as tolerant) and cluster a in trial series 2 (susceptible). Because of the quantitative inheritance of CLS resistance, there are no sharp borderlines between the variety groups. The differences between the datasets are probably due to the different varieties tested and to more severe CLS epidemics in 2014–2016 than in 2009–2011. At highly infested environments, mean DS_end_ was 6.4 in 2014–2016 and 5.8 in 2009–2011 (data not shown). Variety 1, which was the only one tested in both series also showed a higher DS_end_ under high infection in the more recent trial series than in the older one (6.7 vs. 5.8) and a higher relative yield loss (8.2 vs. 6.7%).

The comparison of both datasets is limited by the fact that they origin from different years. For a comparison of older and newer varieties, they should ideally be grown in the same trials (Loel et al., 2014), but this is not possible for a high number of trial sites and varieties. Nevertheless, the data show clearly that the yield penalty of resistant varieties under low infection has disappeared in the varieties currently on the German market. Even regular fungicide applications did not improve the relative competiveness of the susceptible varieties. This supports the assumption by Gummert et al. (2015) that a new generation of resistant varieties is able to catch up with susceptible ones under low infection.

### Economic Performance

In our study, revenue varied by up to € 242 among varieties, direct costs by up to € 52 and operating cost by up to € 12 (data not shown). Variety was thus the key factor for revenue less direct and operating costs as an indicator of economic performance and explains why beet growers choose varieties according to their yield performance (Manthey and Ladewig, 2009).

Under low infection, revenue less direct and operating costs of the variety clusters was close. The greatest difference among varieties was ca. € 150 with both varieties belonging to the susceptible cluster. Under low infection, variety reaction to CLS is thus of lower importance than yield potential. Under high infection, all resistant varieties reached higher revenue less direct and operating costs than susceptible and tolerant ones. Beet growers should thus choose resistant varieties for two reasons: first, tolerant and susceptible varieties show higher yield losses even with fungicide application as also shown by Mittler et al. (2004). Second, resistant varieties usually reach the threshold for fungicide application later than tolerant and susceptible ones. This extends the period for fungicide application and may permit to save at least one spraying (Wolf et al., 1998; Kaiser et al., 2007). Due to higher revenues and the assumption that one fungicide application can be skipped, economic advantage of the resistant varieties was € 152 and € 162 compared to susceptible and tolerant varieties, respectively, which yielded similarly when fungicide was applied.

The difference in revenue less direct and operating costs among fungicide levels indicates whether fungicide application makes economic sense. This is the case when the extra earnings are beyond the fungicide application costs (Lang, 2005). It has to be considered that the yield data for this comparison originate from field trials with the aim to keep the fungicide treated plots as healthy as possible. The cost calculation, however, was based on fungicide applications according to the threshold system. Revenue of fungicide application could thus have been overestimated or costs underestimated, respectively (Kaiser et al., 2007). Under high infection, the mean difference of extra earnings and extra cost was € 163 and is thus most likely economical, even if a certain inaccuracy is supposed.

### Consequences for Integrated Pest Management

White sugar yield was mainly influenced by environment and fungicide treatment. Variety had a minor effect, but it increased at environments with high infection of CLS. Beet growers can hardly influence the environmental conditions driving CLS epidemics such as temperature and humidity, but they determine variety and fungicide strategy. The aim of integrated pest management is to reduce fungicide use and to control fungal diseases by other means as far as possible (EU, 2009). Our results indicate that sugar beet with resistance traits toward *C. beticola* can be one of these means. The current resistant varieties caught up with susceptible ones under low disease pressure and there is thus no longer reason to prefer susceptible varieties and to rely on fungicide applications when CLS might occur. This offers opportunities to increase eco efficiency of sugar beet production in terms of fungicide use (Wießner et al., 2010).

By contrast to the resistant varieties, tolerant varieties had no economic advantage over susceptible ones. Under high infection, WSY was the same in the susceptible and tolerant variety clusters when fungicide was applied. Furthermore, it is unlikely that beet growers will skip a fungicide application on tolerant varieties due to the high DS they express. Following the current threshold system for fungicide application (Wolf and Verreet, 2002; Lang, 2005), the tolerant varieties will thus not contribute to the reduction of fungicide use and they will not increase revenue less direct and operating costs compared to susceptible ones under high infection either. Further studies have thus to assess the importance of resistant/tolerant varieties for integrated pest management in terms of variety specific control thresholds, treatment index (i.e., intensity of fungicide use; Sattler et al., 2007) and management of fungicide resistance.

Gummert et al. (2015) demonstrated that omitting the final fungicide application of two or three applications following the threshold system (Wolf and Verreet, 2002; Lang, 2005) had no effect on WSY independently of the variety type. They pointed out that this advantage has to be weighed against the risk of increasing inoculum potential and stronger epidemics in the following year (Pringas and Märländer, 2004; Khan et al., 2008). Even if the necessary cropping interval of 2–3 years (Windels et al., 1998) is kept, this may concern neighboring fields. Resistant varieties, which delay epidemic development and reduce spore yield (Weiland and Koch, 2004), might nevertheless reduce inoculum potential as well. This has to be assessed in further studies. Moreover, as reduced efficacy of fungicides in relation to their mode of action (Varrelmann and Märländer, 2017) is increasingly observed in commercial practice in central Europe (e.g., Kempl, 2017; Zellner, 2017), resistant varieties may contribute to inhibit this development by reduced fungicide application.

## Conclusion

The older resistant varieties tested in 2009–2011 yielded 2–4% lower than susceptible ones under low infection or healthy conditions. By contrast, the newer resistant varieties tested in 2014–2016 yielded higher than susceptible ones under high infection and showed no yield penalty under low infection or healthy conditions. It can thus be assumed that this new generation of resistant varieties will gain acceptance among growers. It has to be studied in more detail, but there is a realistic chance that these varieties will require less fungicide application than susceptible ones. Resistant varieties will thus enhance both economic and ecological efficiency of sugar beet production, especially under high infection of CLS.

## Author Contributions

BM and CK planned and supervised the study. JV and CK analyzed the field data. JV and CH made the economical evaluation of the data. CK and JV wrote the manuscript with support from BM and CH.

## Conflict of Interest Statement

The authors declare that the research was conducted in the absence of any commercial or financial relationships that could be construed as a potential conflict of interest.
